# Supplementary material for: Enhancing site selection strategies in clinical trial recruitment using real-world data modeling
Source: PLoS One. 2024 Mar 11;19(3):e0300109. doi: 10.1371/journal.pone.0300109 (PMC10927105; doi:10.1371/journal.pone.0300109)
Supplement: S1 File — (DOCX) [file pone.0300109.s001.docx]

| **Indication** | **Study list** |
| --- | --- |
| Inflammatory bowel disease (IBD) | NCT03466411, NCT03105128, NCT03105102, NCT03345836, NCT03345849, NCT03345823, NCT03006068, NCT03653026, NCT02819635, NCT01536418, NCT03464136, NCT03440385, NCT01620255, NCT02531113, NCT02407236, NCT02100696, NCT03566823, NCT01369355, NCT03782376, NCT03934216, NCT01470612, NCT03518086, NCT02065570, NCT03926130, NCT02394028, NCT00552058, NCT01287897, NCT02531126, NCT03104413, NCT03440372, NCT03259308, NCT02435992, NCT03395184, NCT01277666, NCT03524092, NCT01659138, NCT01294410, NCT02891226, NCT02365649, NCT02589665NCT01240915, NCT01393626, NCT04090411, NCT02065622, NCT00349752, NCT01458574, NCT01458951, NCT01751152, NCT02165215, NCT02958865, NCT02782663, NCT02620046, NCT03662542, NCT02031276, NCT00410410, NCT00406653, NCT04033445, NCT02877134, NCT02171429, NCT01393899,NCT01771809, NCT03482635, NCT03559517, NCT01696396, NCT01647516, NCT01465763, NCT01694485, NCT05197049, NCT03695185, NCT01470599, NCT03398135, NCT04102111, NCT02871635, NCT02903966, NCT01545050, NCT04677179, NCT03519945, NCT01714726, NCT01150890, NCT03558152,NCT01199302, NCT01466374, NCT02163759, NCT02185014, NCT01203631, NCT01336465, NCT02840721 |
| Multiple Myeloma (MM) | NCT00689936, NCT02726581, NCT01478048, NCT02159365, NCT02874742, NCT01335399, NCT01239797, NCT02195479, NCT02312258, NCT02654990, NCT03277105, NCT02477891, NCT02252172, NCT04557098, NCT03848845, NCT03525678, NCT03652064, NCT03301220, NCT02076009, NCT01985126, NCT01080391, NCT02755597, NCT01818752, NCT01568866, NCT03544281, NCT01734928, NCT01441973, NCT03412565, NCT01602224, NCT00546780, NCT04649359, NCT02719613, NCT02612779, NCT02136134, NCT02316106, NCT02807454, NCT01850524, NCT02181413, NCT02951819, NCT01946477,NCT00401843, NCT04181827, NCT04126200, NCT03104270, NCT02990338, NCT02412878, NCT01484275, NCT02654132, NCT03275285, NCT03000452, NCT00555906, NCT02899052, NCT01524978, NCT04162210, NCT04246047, NCT03567616, NCT03030261, NCT00718419, NCT00574288, NCT02903381,NCT04484623, NCT03158688, NCT03170882, NCT03289299, NCT01311687 |
