# Supplementary material for: Enhancing site selection strategies in clinical trial recruitment using real-world data modeling
Source: PLoS One. 2024 Mar 11;19(3):e0300109. doi: 10.1371/journal.pone.0300109 (PMC10927105; doi:10.1371/journal.pone.0300109)
Supplement: S2 File — (DOCX) [file pone.0300109.s002.docx]

**Inflammatory bowel disease (IBD)**

| **Criteria** | **Values** |
| --- | --- |
| Diagnosis codes | K50* K51* |
| Drug codes | ‘00074433902’, ‘00074379902’, ‘00074055402’, ‘00074024302’, ‘00074433906’, ‘00074433901’, ‘00074433907’, ‘58118994802’, ‘00074937402’, ‘00074012403’, ‘00074061602’, ‘00074153903’, ‘00074055471’, ‘00074433971’, ‘00074634702’, ‘00074081702’, ‘00074012474’, ‘54868482200’, ‘00074379903’, ‘00074254003’, ‘00074006702’, ‘00074379906’, ‘00074379901’, ‘00074433974’, ‘00074379701’, ‘00074055474’, ‘62195040001’, ‘00074937471’, ‘00074024371’, ‘71202000202’, ‘00074379702’, ‘00074379971’, ‘00074254001’, ‘00074061671’, ‘00074055401’, ‘00074012401’, ‘50090448700’, ‘62195040101’, ‘50090353000’, ‘00074433973’, ‘00074055404’, ‘00074055406’, ‘00074055473’, ‘00074012473’, ‘57894006103’, ‘57894006003’, ‘57894005427’, ‘57894006002’, ‘57894006004’, ‘57894006102’, ‘57894006104’, ‘57894005416’, ‘57894006001’, ‘64764030020’, ‘57894003001’, ‘00069080901’, ‘00006430502’, ‘59784003001’, ‘00006430501’, ‘55513067001’, ‘32228000101’, ‘64029910001’, ‘64029910002’, ‘00409259220’, ‘50474071079’, ‘50474070062’, ‘50474071081’, ‘50474071080’, ‘50474070061’, ‘43284001002’, ‘57894007002’, ‘57894035001’, ‘57894007001’, ‘57894007102’, ‘57894007101’, ‘57894035089’, ‘57894007089’, ‘57894007189’, ‘57894007090’, ‘57894007190’, ‘66914105002’, ‘66914105001’, ‘00069100101’, ‘00069050130’, ‘00069100201’, ‘00069050114’, ‘00069050230’, ‘62157025701’, ‘62157038301’, ‘63539001202’, ‘00069102901’, ‘00069102902’, ‘00069100103’, ‘63539001602’, ‘62157061701’, ‘00069100202’, ‘00069100203’, ‘00069100102’, ‘63539050130’, ‘63539050230’, ‘63539050114’, ‘64406000801’, ‘59075073015’, ‘64406073001’, ‘64406073002’, ‘00409980015’ |
| Procedure codes | ‘J9145’, ‘C9476’, ‘J9144’, ‘J9176’, ‘C9477’, ‘J9047’, ‘J9041’, ‘J9044’, ‘J1094’, ‘J1095’, ‘J1096’, ‘J1100’, ‘J7312’, ‘J7637’, ‘J7638’, ‘J8540’, ‘30230G1’, ‘30230G2’, ‘30230G3’, ‘30230G4’, ‘30230X1’, ‘30230X2’, ‘30230X3’, ‘30230X4’, ‘30230Y1’, ‘30230Y2’, ‘30230Y3’, ‘30230Y4’, ‘30233G1’, ‘30233G2’, ‘30233G3’, ‘30233G4’, ‘30233X1’, ‘30233X2’, ‘30233X3’, ‘30233X4’, ‘30233Y1’, ‘30233Y2’, ‘30233Y3’, ‘30233Y4’, ‘30240G1’, ‘30240G2’, ‘30240G3’, ‘30240G4’, ‘30240X1’, ‘30240X2’, ‘30240X3’, ‘30240X4’, ‘30240Y1’, ‘30240Y2’, ‘30240Y3’, ‘30240Y4’, ‘30243G1’, ‘30243G2’, ‘30243G3’, ‘30243G4’, ‘30243X1’, ‘30243X2’, ‘30243X3’, ‘30243X4’, ‘30243Y1’, ‘30243Y2’, ‘30243Y3’, ‘30243Y4’, ‘30250G1’, ‘30250X1’, ‘30250Y1’, ‘30253G1’, ‘30253X1’, ‘30253Y1’, ‘30260G1’, ‘30260X1’, ‘30260Y1’, ‘30263G1’, ‘30263X1’, ‘30263Y1’, ‘S2142',’'3‘230AZ',’'3‘233AZ',’'3‘240AZ',’'3‘243AZ',’’38206’, ‘38232’, ‘38241’, ‘S2150’, ‘38230’, ‘38205’, ‘38240’, ‘38242’ |
| Specialty | Gastro* |
| Indications | IBD, Crohn’s disease, Ulcerative Colitis |
| Minimum patient age | 18 |
| Start & end date | 2020-01-01 until 2022-12-31 |

**Multiple Myeloma (MM)**

| **Criteria** | **Values** |
| --- | --- |
| Diagnosis codes | C90* C91* |
| Drug codes | ‘57894050205’, ‘57894050220’, ‘57894050301’, ‘3452211’, ‘3229111’, ‘76075010101’, ‘76075010201’, ‘76075010301’, ‘63020007801’, ‘63020007802’, ‘63020007901’, ‘63020007902’, ‘63020008001’, ‘63020008002’, ‘63020023001’, ‘63020023002’, ‘63020039001’, ‘63020039002’, ‘63020040001’, ‘63020040002’, ‘59572050100’, ‘59572050121’, ‘59572050200’, ‘59572050221’, ‘59572050300’, ‘59572050321’, ‘59572050400’, ‘59572050421’, ‘66484240500’, ‘66484240501’, ‘66484241000’, ‘66484241001’, ‘66484415000’, ‘66484415001’, ‘66484415201’, ‘66484425000’, ‘66484425201’, ‘59572040200’, ‘59572040228’, ‘59572040500’, ‘59572040528’, ‘59572040530’, ‘59572041000’, ‘59572041028’, ‘59572041030’, ‘59572041500’, ‘59572041521’, ‘59572042000’, ‘59572042021’, ‘59572042500’, ‘59572042521’, ‘59572042525’, ‘63069030501’, ‘63069031002’, ‘63069031503’, ‘63069032504’, ‘43593040502’, ‘43593041002’, ‘43593041502’, ‘43593042502’, ‘64144099191’, ‘64144099292’, ‘64144099393’, ‘64144099494’, ‘59572010592’, ‘59572010593’, ‘59572010594’, ‘59572020514’, ‘59572020517’, ‘59572020594’, ‘59572020597’, ‘59572021015’, ‘59572021028’, ‘59572021095’, ‘59572021513’, ‘59572021593’, ‘59572022016’, ‘59572022096’, ‘59572220594’, ‘63069020501’, ‘63069021002’, ‘63069021505’, ‘63069022003’, ‘63069063069’, ‘5957210511’,  ‘43598086560’, ‘63323072110’, ‘10130004901’, ‘10130004901’, ‘63020004901’, ‘63020004902’, ‘63020004903’, ‘63020004904’, ‘72237010101’, ‘72237010102’, ‘72237010103’, ‘72237010104’, ‘72237010105’, ‘72237010106’, ‘72237010107’, ‘72237010111’, ‘72237010112’, ‘72237010113’, ‘72237010114’, ‘72237010115’, ‘72237010116’, ‘72237010117’, ‘72237010202’, ‘72237010206’, ‘72237010207’, ‘72237010212’, ‘72237010216’, ‘72237010217’, ‘72237010305’, ‘72237010315’, ‘72237010401’, ‘72237010411’ |
| Procedure codes | ‘J9145’, ‘C9476’, ‘J9144’, ‘J9176’, ‘C9477’, ‘J9047’, ‘J9041’, ‘J9044’, ‘J1094’, ‘J1095’, ‘J1096’, ‘J1100’, ‘J7312’, ‘J7637’, ‘J7638’, ‘J8540’, ‘30230G1’, ‘30230G2’, ‘30230G3’, ‘30230G4’, ‘30230X1’, ‘30230X2’, ‘30230X3’, ‘30230X4’, ‘30230Y1’, ‘30230Y2’, ‘30230Y3’, ‘30230Y4’, ‘30233G1’, ‘30233G2’, ‘30233G3’, ‘30233G4’, ‘30233X1’, ‘30233X2’, ‘30233X3’, ‘30233X4’, ‘30233Y1’, ‘30233Y2’, ‘30233Y3’, ‘30233Y4’, ‘30240G1’, ‘30240G2’, ‘30240G3’, ‘30240G4’, ‘30240X1’, ‘30240X2’, ‘30240X3’, ‘30240X4’, ‘30240Y1’, ‘30240Y2’, ‘30240Y3’, ‘30240Y4’, ‘30243G1’, ‘30243G2’, ‘30243G3’, ‘30243G4’, ‘30243X1’, ‘30243X2’, ‘30243X3’, ‘30243X4’, ‘30243Y1’, ‘30243Y2’, ‘30243Y3’, ‘30243Y4’, ‘30250G1’, ‘30250X1’, ‘30250Y1’, ‘30253G1’, ‘30253X1’, ‘30253Y1’, ‘30260G1’, ‘30260X1’, ‘30260Y1’, ‘30263G1’, ‘30263X1’, ‘30263Y1’, ‘S2142',’'3‘230AZ',’'3‘233AZ',’'3‘240AZ',’'3‘243AZ',’’38206’, ‘38232’, ‘38241’, ‘S2150’, ‘38230’, ‘38205’, ‘38240’, ‘38242’ |
| Specialty | Onco* |
| Indications | Multiple myeloma |
| Minimum patient age | 18 |
| Start & end date | 2020-01-01 until 2022-12-31 |
