# Supplementary material for: Enhancing site selection strategies in clinical trial recruitment using real-world data modeling
Source: PLoS One. 2024 Mar 11;19(3):e0300109. doi: 10.1371/journal.pone.0300109 (PMC10927105; doi:10.1371/journal.pone.0300109)
Supplement: S3 File — (DOCX) [file pone.0300109.s003.docx]

| **Indication** | **Covariates** |
| --- | --- |
| Inflammatory bowel disease (IBD) | Enrollment months Site open year,  EWMA of patients per site per month in the last 5 year,  EWMA rank in enrolled patients in the last 5 year,  Sum of number of enrolled patients in the last 5 year,  Median of number of enrolled patients in the last 5 year,  Number of publications,  Number of claims,  Number of specialists,  Number of treated specialists,  Number of treating physicians,  Number of visits,  Number of ongoing trials,  Patient flow difference,  Trial experience,  Visits per patient |
| Multiple Myeloma (MM) | Enrollment months,  Site open year,  Median enrollment rate in the last 5 year,  EWMA of patients per site per month in the last 5 year,  EWMA rank in enrolled patients in the last 5 year,  Median of number of enrolled patients in the last 5 year,  Number of publications,  Number of claims,  Number of physicians,  Number of patients,  Number of specialists,  Number of treated specialists,  Number of treating physicians,  Number of visits,  Number of ongoing trials,  Patient flow difference,  Site flow difference,  Visits per patient |
