# Supplementary material for: Enhancing site selection strategies in clinical trial recruitment using real-world data modeling
Source: PLoS One. 2024 Mar 11;19(3):e0300109. doi: 10.1371/journal.pone.0300109 (PMC10927105; doi:10.1371/journal.pone.0300109)
Supplement: S4 File — (DOCX) [file pone.0300109.s004.docx]

**Constant parameters**

| **Parameter name** | **Value** |
| --- | --- |
| objective | count: poisson |
| eval_metric | poisson-nloglik |
| tree_method | gbtree |

**Tunable parameters**

| **Parameter name** | **Value** |
| --- | --- |
| max_depth | [1, 7] |
| min_child_weight | [32, 256] |
| n_estimators | [32, 4084] |
| reg_lambda | [64, 1000] |
| alpha | [0.00001, 1.0] |
| subsample | [0.2, 0.7] |
| colsample_bytree | [0.2, 0.7] |
| colsample_bylevel | [0.2, 0.7] |
| eta | [0.0001, 0.5] |
| gamma | [0.0001, 0.01] |
| grow_policy | [depthwise, lossguide] |

**Final IBD parameters**

| **Parameter name** | **Value** |
| --- | --- |
| max_depth | 2 |
| min_child_weight | 150 |
| n_estimators | 1611 |
| reg_lambda | 719 |
| alpha | 5.66e-05, |
| subsample | 0.62 |
| colsample_bytree | 0.67 |
| colsample_bylevel | 0.42 |
| eta | 0.028 |
| gamma | 0.0005 |
| grow_policy | lossguide |

**Final MM parameters**

| **Parameter name** | **Value** |
| --- | --- |
| max_depth | 2 |
| min_child_weight | 192 |
| n_estimators | 315 |
| reg_lambda | 383 |
| alpha | 0.0009 |
| subsample | 0.53 |
| colsample_bytree | 0.49 |
| colsample_bylevel | 0.67 |
| eta | 0.04 |
| gamma | 0.006 |
| grow_policy | depthwise |
