# Supplementary material for: Enhancing site selection strategies in clinical trial recruitment using real-world data modeling
Source: PLoS One. 2024 Mar 11;19(3):e0300109. doi: 10.1371/journal.pone.0300109 (PMC10927105; doi:10.1371/journal.pone.0300109)
Supplement: S1 Table — (DOCX) [file pone.0300109.s005.docx]

| **Indication(s)** | **Number of benchmark studies** | **Number of US sites** | **Number of study site combinations** | **Patients per site (p/s) mean (standard deviation)** | **Number of sites per benchmark study mean (standard deviation)** |
| --- | --- | --- | --- | --- | --- |
| Inflammatory bowel disease (IBD) | 87 | 496 | 2046 | 2.54 (3.04) | 23.5  (20.5) |
| Multiple Myeloma (MM) | 65 | 302 | 718 | 3.48 (5.65) | 11.05 (12.25) |
