# Supplementary material for: Enhancing site selection strategies in clinical trial recruitment using real-world data modeling
Source: PLoS One. 2024 Mar 11;19(3):e0300109. doi: 10.1371/journal.pone.0300109 (PMC10927105; doi:10.1371/journal.pone.0300109)
Supplement: S2 Table — (DOCX) [file pone.0300109.s006.docx]

| **Indication** | **Model** | **Train R2** | **Train Spearman correlation coefficient** | **Train RMSE** | **Train MAE** | **Train Top 30% AUC** | **Train Bottom 30% AUC** |
| --- | --- | --- | --- | --- | --- | --- | --- |
| IBD | Median Baseline | - | - | 2.43 | 1.71 | 0.50 | 0.50 |
|  | Site Baseline | - | 0.08 | 2.43 | 2.08 | 0.53 | 0.55 |
|  | Linear Model | 0.15 | 0.40 | 2.20 | 1.66 | 0.72 | 0.78 |
|  | Random  Forest | 0.28 | 0.53 | 2.03 | 1.53 | 0.80 | 0.88 |
|  | XGBoost | 0.28 | 0.50 | 2.02 | 1.52 | 0.79 | 0.87 |
| MM | Median Baseline | - | - | 3.68 | 2.30 | 0.50 | 0.50 |
|  | Site Baseline | - | 0.22 | 4.28 | 2.93 | 0.60 | 0.67 |
|  | Linear Model | 0.14 | 0.37 | 3.20 | 2.35 | 0.72 | 0.77 |
|  | Random Forest | 0.24 | 0.48 | 3.02 | 2.20 | 0.78 | 0.86 |
|  | XGBoost | 0.20 | 0.45 | 3.08 | 2.20 | 0.77 | 0.79 |
