# Supplementary material for: Enhancing site selection strategies in clinical trial recruitment using real-world data modeling
Source: PLoS One. 2024 Mar 11;19(3):e0300109. doi: 10.1371/journal.pone.0300109 (PMC10927105; doi:10.1371/journal.pone.0300109)
Supplement: Attachment — Submitted filename: Response to Reviewers.docx [file pone.0300109.s007.docx]

Lars Hulstaert
Johnson & Johnson GmbH

Johnson & Johnson Platz 2
41470 Neuss
Germany
lhulsta1@its.jnj.com

**To: PLOS One**

Dear Editor,

I hope this letter finds you well. I appreciate the time and effort invested by the editorial team and reviewers in evaluating my Research Article titled "Enhancing Site Selection Strategies in Clinical Trial Recruitment Using Real-World Data Modeling" for the PLOS One Journal. I am grateful for the feedback provided, and I would like to submit a rebuttal addressing the comments raised during the review process.

**Reviewer 1 Comments**

| **Comment** | **Response** |
| --- | --- |
| Definition of patient cohorts for benchmark studies: Suppl. file S2 indicates that multiple clinical studies were used as reference for the identified patient cohorts. Please comment on how the cohort inclusion criteria for the presented 'experiments' were derived from the inclusion criteria of these clinical studies. Presumably, inclusion criteria were not identical for all clinical studies, how was consensus reached for the experiments? | Updated line 166: Inclusion criteria of benchmark studies are used to define a superset of relevant diagnosis, drugs, and procedures codes. These codes define a patient cohort that represents the broad patient population that is eligible for the benchmark studies. |
| Mismatch between RWD cohort observation period and recruitment data: "As such, the cohort observation period is used instead to characterize the real-world clinical practice of a site, and it is assumed to remain constant over time". Please discuss the potential biases that may be introduced by estimating site level covariates for the recruitment into studies performed much earlier (about 15-20 years, 1990 onwards) from rather recent RWD (2016 onwards). | Updated line line 212: The variability in yearly calculations of the site level RWD covariates across the available data is sufficiently small, allowing them to be approximated as constant when averaged across the cohort observation period. Before 2016 it is not possible to validate this hypothesis which has the potential to introduce data bias in RWD covariates for studies conducted before 2016. |
| Manual covariate selection: What thresholds have been applied and which covariates were selected/removed? Does fig 4 show only the selected covariates? | Updated line 269: Covariates with a variable importance, as defined by the covariate mean SHAP value, that is below 0.005 are removed from the covariate set. For each model, the selected set of covariates is defined in S4 File, which is a subset of the full set of covariates described in Table 3.    Fig 4 description is adapted to “Covariate importance of the selected covariates for the XGBoost models” |
| Authors state in the discussion that 'The non-linear model performance significantly improves the ability to rank the sites by expected enrollment ...', however, the methods section indicates that statistical significance testing has been performed on the MAE which is not a direct measure of ranking performance. Please explain. | Updated line 306 and 320 to reflect the type of statistical testing that was applied. |
| Fig 1-3: Please indicate the unit of 'frequency' in figs 1-3. 'Studies per year' in fig 1? But 'number of studies' in fig 2 and fig 3? | Descriptions of Fig 1-3 are adapted (line 138 to 145):  Fig 1. Overview of number of benchmark studies across phase and indication. An overview of the number of benchmark studies across study phase and study indication for resp. IBD and MM.  Fig 2. Overview of number of patients enrolled and number of enrollment months across study-site combinations. An overview of the number of patients enrolled and number of enrollment months per site across the benchmark studies for resp. IBD and MM.  Fig 3. Overview of number of sites across US states and open year. An overview of number of sites across US states and site open year for resp. IBD and MM. |
| Table 4: The reported results are labeled as 'Test'. Presumably this refers to the 20% of the 80/20 split mentioned in line 217. Please clarify the nomenclature. It would be interesting to see how robust these performance estimates are for different sample splits. | Updated line 224: We use a random train (80%) and test (20%) data split at site level to avoid the potential of a data distribution bias and corresponding impact on model generalization capabilities. |
| Table S6 does not contain train model performances as mentioned in line 265. | Updated Table S6 to contain the train model performance. |

Reviewer 2 Comments

| **Comment** | **Response** |
| --- | --- |
| Since there are many non-linear models besides XGBoost, it would be interesting to add a few more models to test how different algorithms perform in the two indications. Since the training/test data are ready, this seems straightforward. But if there are practical reasons why other algorithms were not selected, you may simply add the explanation. | Updated Table 4 with results for RandomForest experiments.  Updated line 253: For each exercise, a linear Poisson regression model and two non-linear machine learning models, a RandomForest and an XGBoost model (v.1.7.2), are trained and compared with the median and site baseline.  We considered other non-linear models but didn’t observe a significant difference in performance. |

**Updated Funding Statement**

The authors received no specific funding for this work.

All authors of this manuscript are full-time salaried employees of Janssen Research & Development, a pharmaceutical company of Johnson & Johnson. The funder, Janssen Research & Development, provided support in the form of salaries for all authors but did not have any additional role in the study design, data collection and analysis, decision to publish, or preparation of the manuscript. The specific roles of the authors are articulated in the ‘author contributions’ section.

**Updated Competing Interest Statement**

All authors are employees of Janssen Research and Development, a unit of Johnson and Johnson family of companies. The work on this study was part of their employment. All authors hold pension rights from the company and own stock options.

This does not alter our adherence to PLOS ONE policies on sharing data and materials.

I have reviewed each comment, and I believe the revisions made strengthen the manuscript.

I would like to express my gratitude for the opportunity to submit this revised manuscript for reconsideration. I believe the revisions adequately address the concerns raised, and I am confident that the manuscript is now in line with the standards and objectives of PLOS One.

Thank you for considering our work for inclusion in your journal. We look forward to your feedback.

Sincerely,

Lars Hulstaert
Johnson & Johnson
